# Abscisic acid synergizes with sucrose to enhance grain yield and quality of rice by improving the source-sink relationship

**DOI:** 10.1186/s12870-019-2126-y

**Published:** 2019-11-27

**Authors:** Tingting Chen, Guangyan Li, Mohammad Rezaul Islam, Weimeng Fu, Baohua Feng, Longxing Tao, Guanfu Fu

**Affiliations:** 1 National Key Laboratory of Rice Biology, China National Rice Research Institute, Hangzhou, 310006 People’s Republic of China; 2 Department of Agricultural Extension, Ministry of Agriculture, Dhaka, 1215 Bangladesh

**Keywords:** Rice (*Oryza sativa* L.), Abscisic acid, Sucrose, Grain yield and quality, Assimilates allocation, Sugar metabolism

## Abstract

**Background:**

Abscisic acid (ABA) and sucrose act as molecular signals in response to abiotic stress. However, how their synergy regulates the source-sink relationship has rarely been studied. This study aimed to reveal the mechanism underlying the synergy between ABA and sucrose on assimilates allocation to improve grain yield and quality of rice. The early indica rice cultivar Zhefu802 was selected and planted in an artificial climate chamber at 32/24 °C (day/night) under natural sunlight conditions. Sucrose and ABA were exogenously sprayed (either alone or in combination) onto rice plants at flowering and 10 days after flowering.

**Results:**

ABA plus sucrose significantly improved both the grain yield and quality of rice, which was mainly a result of the higher proportion of dry matter accumulation and non-structural carbohydrates in panicles. These results were mainly ascribed to the large improvement in sucrose transport in the sheath-stems in response to the ABA plus sucrose treatment. In this process, ABA plus sucrose significantly enhanced the contents of starch, gibberellic acids, and zeatin ribosides as well as the activities and gene expression of enzymes involved in starch synthesis in grains. Additionally, remarkable increases in trehalose content and expression levels of *trehalose-6-phosphate synthase1*, *trehalose-6-phosphate phosphatase7*, and *sucrose non-fermenting related protein kinase 1A* were also found in grains treated with ABA plus sucrose.

**Conclusion:**

The synergy between ABA and sucrose increased grain yield and quality by improving the source-sink relationship through sucrose and trehalose metabolism in grains.

## Background

Rice (*Oryza sativa* L.) is the main daily dietary food source of a large share of the human population, and the starch and protein in the grain supports the world’s population [1]. Accordingly, as grain production needs to support an ever-increasing number of people combined with inevitable deterioration in the environment, crop yield and grain quality of rice need to be improved to guarantee food safety worldwide. It has been acknowledged that carbohydrate allocation concerning the source-sink relationship is essential for improving the yield and quality of rice, which is affected by environmental factors, including soil drought, nutritional deficiency, and heat stress [2–4]. Therefore, much attention has been focused on promoting the source-to-sink transport of photoassimilates to enhance the grain yield and quality of rice by exogenous plant growth regulation, including abscisic acid (ABA) and sucrose.

ABA is a central phytohormone that is involved in the biological processes of seed germination, root differentiation, fruit maturation, stomatal closure, inhibition of photosynthesis, and leaf senescence in response to many abiotic stresses [5]. It has been reported that the abundance of aquaporins and cellular hydraulic conductivity within root tissues can be altered by exogenous application of ABA in barley [6]. Furthermore, an exogenous application of ABA stimulates the expression of a number of genes that are responsible for ABA biosynthesis, accelerates recovery of the photosynthetic system destroyed by drought stress in upland rice, and enhances cold tolerance of wheat during the grain filling stage [7, 8]. Recent studies have also suggested its regulatory role in sugar metabolism and translocation, which further regulate dry matter accumulation and grain filling [9, 10]. For instance, an appropriate concentration of exogenously applied ABA enhances the activity of sucrose synthase (SUS) and the expression of genes involved in starch synthesis; thus, improving the grain yield of rice [11, 12]. In response to high temperature conditions, ABA increases the concentrations of soluble sugar, starch, and non-structural carbohydrate (NSC), enhances gene expression of heat shock proteins and proteins participating in sugar transport and conversion, and improves the activities of antioxidases and adenosine triphosphate content in rice plant spikelets [10]. These results suggest that exogenous application of ABA could enhance sucrose transport and accelerate sucrose-to-starch conversion, thus safeguarding carbohydrate metabolism and energy homeostasis against external stress [10]. Therefore, a possible interaction may exist between sucrose and ABA in mediating the activity and expression of SUS during the grain filling stage in rice [11].

Sucrose is an energy source of plants and a signaling molecule that regulates plant development [13, 14]. It has been reported that sucrose exerts an immediate effect on nitrogen assimilation and transport as well as balance of carbon-nitrogen metabolism. In some dicotyledonous plants and *Arabidopsis thaliana* adapted to the dark, an increase in gene transcription level of nitrate reductase was elicited by sucrose [15, 16]. Sucrose also modulates central regulators of material and energy metabolism, mainly through sucrose non-fermenting related protein kinase 1 (SnRK1). SnRK1 could cause extensive reprogramming of gene transcription and affect plant growth as a central integrator of energy signaling under abiotic stress [17–19]. Sucrose is considered the main signaling molecule involved in mediating the source-sink relationship at the flowering stage of rice [4]. Indeed, the role of sucrose as a signaling molecule in plants is complex, and it may interact with other endogenous or environmental signals, such as ABA. Previous studies have reported that the interaction between ABA and sugars regulates plant reproductive development in response to photoperiod, carbohydrate synthesis, and accumulation during fruit growth [20, 21]. Exogenous glucose increases endogenous ABA levels, as well as the expression of ABA synthesis and signal transduction-related genes [22]. The complex interplay between sugar and hormone signaling has been shown to result in plasticity of plant growth and development [23]. The sugars involved in this process might include sucrose in addition to glucose and fructose. It has been reported that ABA exerts a collaborative role with sucrose during anthocyanin synthesis, while this pathway induced by sucrose is inhibited by additional application of gibberellic acids (GAs) [24]. Sucrose transporters, such as *SUC2* and *SUC4*, are crucial regulatory factors in response to abiotic stresses and that enhance plant tolerance through the ABA signaling pathway, suggesting that there might be cross-talk with sucrose signaling [25]. In addition, sucrose and ABA synergistically increase starch content and adenosine diphosphate glucose pyrophosphorylase (AGPase) activity of the maize endosperm as well as regulate tomato and strawberry fruit ripening, in which the gene involved in the sucrose and ABA signal transduction pathway is the main mediator [26, 27]. These results suggest that the interaction between ABA and sucrose plays a key role in plant development processes, such as grain filling and starch synthesis. However, the synergy between ABA and sucrose functions in source-sink carbohydrate allocation of rice and its effect on grain quality has not been documented.

The source-sink relationship is related to the assimilate distribution between leaves, sheath-stems, and grains. The deposited sucrose in rice grains is the main form of carbohydrate in this process. Sucrose originates from carbon assimilation in leaves and is transported and translocated from photosynthetically active tissues to non-photosynthetic sinks, such as fruits, seeds, and developing tubers [28, 29]. When it reaches these sinks, sucrose is degraded into hexoses and then metabolized via several metabolic and biosynthetic processes, which ultimately lead to the synthesis of starch. However, this process can be disturbed by environmental factors resulting not only in lower spikelet fertility, kernel weight, and grain yield, but also in poorer grain quality. The present study shows that the synergy between ABA and sucrose significantly improved grain yield and quality of rice during the grain filling stage. Thus, the distribution of dry matter weight and NSC, sucrose transport, and metabolic genes, phytohormones, and trehalose metabolism were determined to investigate the role of the crosstalk between ABA and sucrose in the source-sink relationship of rice plants during the flowering and grain filling stages of rice.

## Results

### Effect of ABA and sucrose on grain yield and rice quality

**Fig. 1:**
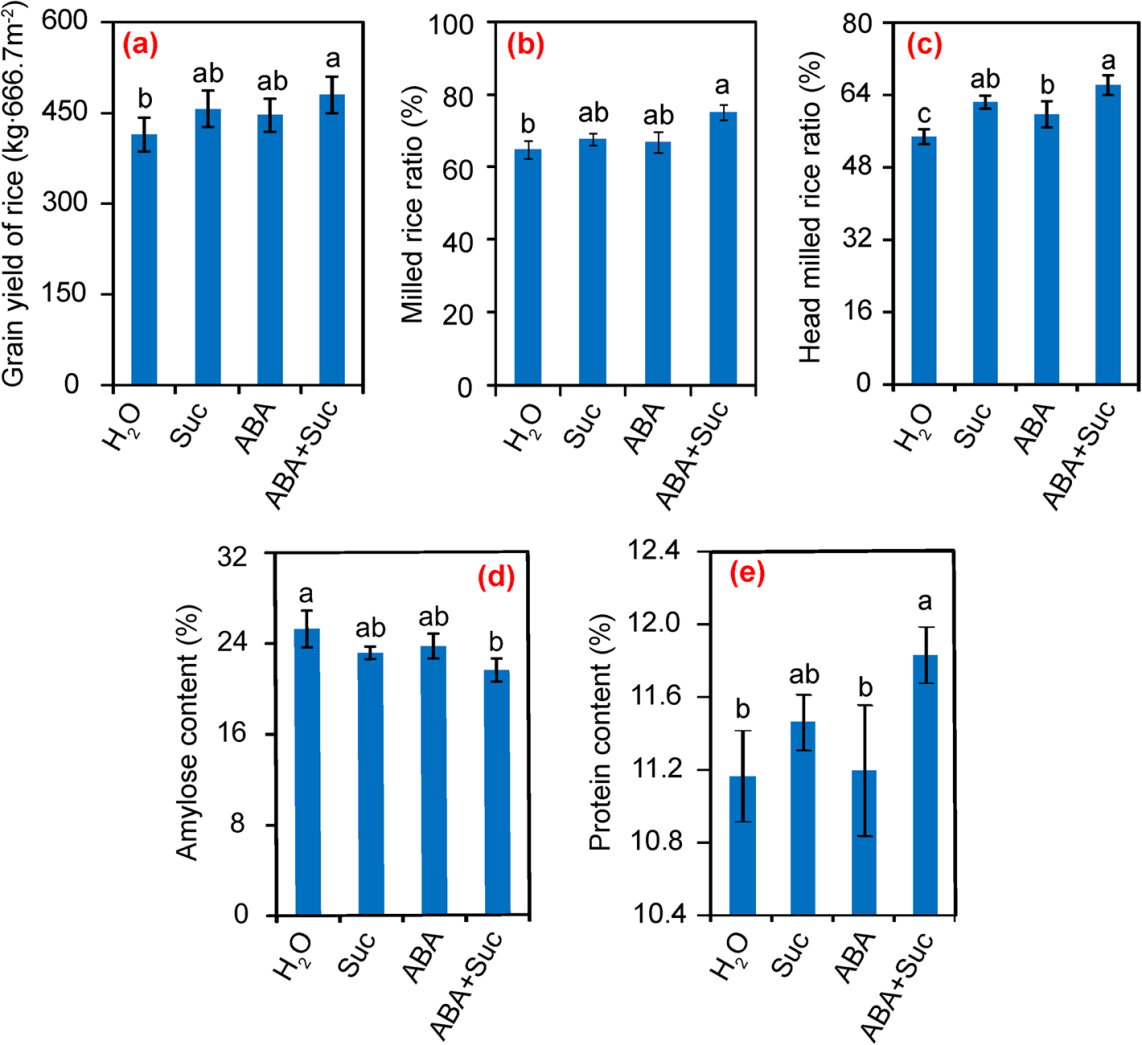
Effect of sucrose and abscisic acid (ABA) alone or in combination on the grain yield and grain quality of rice. **a** Effect of sucrose and ABA alone or in combination on the grain yield; **b**–**e**, effect of sucrose and ABA alone or in combination on the milled rice ratio, head milled rice ratio, amylose content, and protein content. Vertical bars denote standard deviations (*n* = 3). Different letters indicate significant differences between chemical treatments (*P* < 0.05)

Rice plants exogenously sprayed with low sucrose concentrations attained higher grain yields than the control, particularly for the treatments with 0.5% sucrose, which achieved a 13.5% increase in grain yield compared to the control (Additional file 1: Figure S1). Grain yield increased by 9.2% under 10 μmol·L^− 1^ ABA treatment compared to the control, although the difference between them was not significant (Additional file 1: Figure S1). The enhancement of grain yield in response to ABA was eliminated by its inhibitor and the grain yield decreased by 5.7 and 12.9% under the 10 and 100 μmol·L^− 1^ fluridone treatments, respectively (Additional file 1: Figure S1). According to the positive effect of sucrose and ABA on grain yield, rice plants were either sprayed with sucrose and ABA alone or in combination after selecting the optimized concentration. The synergistic effect of ABA and sucrose on grain yield and quality were investigated. Without exception, no significant differences were found in grain yield between the control and the ABA or sucrose alone treatments, but about 10.3 and 7.8% increases were found in rice plants treated with sucrose and ABA, respectively, compared to the control (Fig. 1a). However, a pronounced increase of grain yield by 15.7% was attained by rice plants treated with ABA plus sucrose.

A significant increase in the milled rice ratio, head milled rice ratio, protein content, and a significant decrease in amylose content were detected in plants under the ABA plus sucrose treatment compared to the control (Fig. 1b–e). Thus, grain quality was significantly improved by the ABA plus sucrose treatment, which enhanced milling quality, cooking and eating quality, and nutritional quality. However, no significant differences were found in the other rice quality criteria among the sucrose and ABA treatments (Additional file 2: Table S1).

### Effect of ABA and sucrose on seed-setting rate, pollen tube elongation, grain weight, and grain morphology

**Fig. 2:**
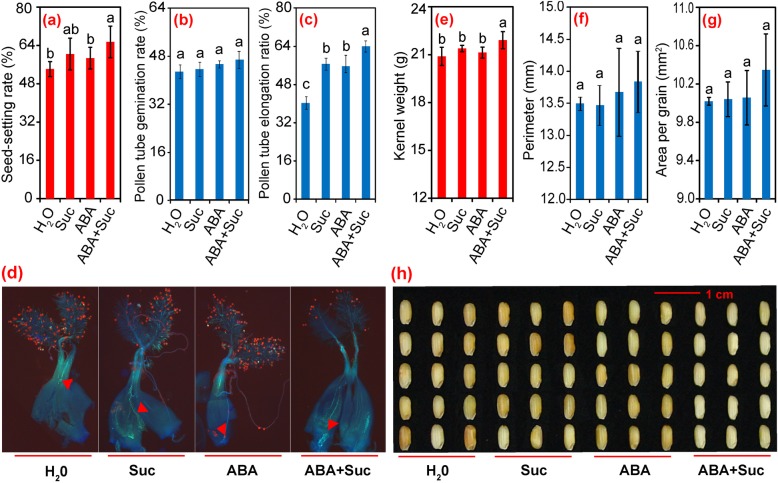
Effect of sucrose and abscisic acid (ABA) alone or in combination on seed-setting rate, pollen tube growth, grain weight, and grain morphology of rice. **a** Effect of sucrose and ABA alone or in combination on the seed-setting rate; **b**, effect of sucrose and ABA alone or in combination on the pollen germination rate; **c** and **d**, effect of sucrose and ABA alone or in combination on pollen tube elongation; **e**, effect of sucrose and ABA alone or combination on kernel weight; **f**–**h**, effect of sucrose and ABA alone or in combination on grain size. Vertical bars denote standard deviations (*n* = 3). Different letters indicate significant differences between chemical treatments (*P* < 0.05)

As the spray treatments were initiated at anthesis and during the grain filling period, the panicle numbers and grain numbers per panicle were barely affected by the sucrose or ABA treatments. Seed-setting rate only significantly increased under the ABA plus sucrose treatment, where increases of about 8.8, 6.4, and 15.9% were observed under the treatments of sucrose, ABA, and ABA plus sucrose, respectively, compared to the control (Fig. 2a).

Pollen tube germination and growth play a crucial role in sexual reproduction of flowering plants before fertilized female germ cells successfully develop from the embryo and endosperm into mature seeds [4]. Therefore, the germination ratio of pollen sticking to the stigma and the status of the pollen tube entering the embryo sac were monitored to investigate the reasons for the different seed-setting rates among the treatments. No significant difference was found in the ratio of germinated pollen numbers between the sucrose, ABA, and ABA plus sucrose treatments, and the control (Fig. 2b). However, pollen tube elongation scarcely occurred in the ovaries of control plants, which resulted in a low ratio of pollen tube elongation (Fig. 2c and d). The pollen tube elongation ratio increased significantly in plants treated with sucrose, ABA, or ABA plus sucrose (in particular by the latter) compared to the control. These results suggest that pollen tube elongation was promoted by ABA plus sucrose, which could contribute to more fertilized spikelets and a higher seed-setting rate (Fig. 2a, c, and d).

Kernel weight of plants treated with ABA plus sucrose was significantly higher (by 4.9%) than the control (Fig. 2e). No significant differences were found among sucrose, ABA, or the ABA plus sucrose treatments in the perimeter or area per grain of brown rice, although slight increases were found in grains treated with ABA plus sucrose compared to the control (Fig. 2f–h). The results show that grain weight was improved by ABA plus sucrose without affecting grain morphology.

### Effect of ABA and sucrose on dry matter accumulation and allocation

**Fig. 3:**
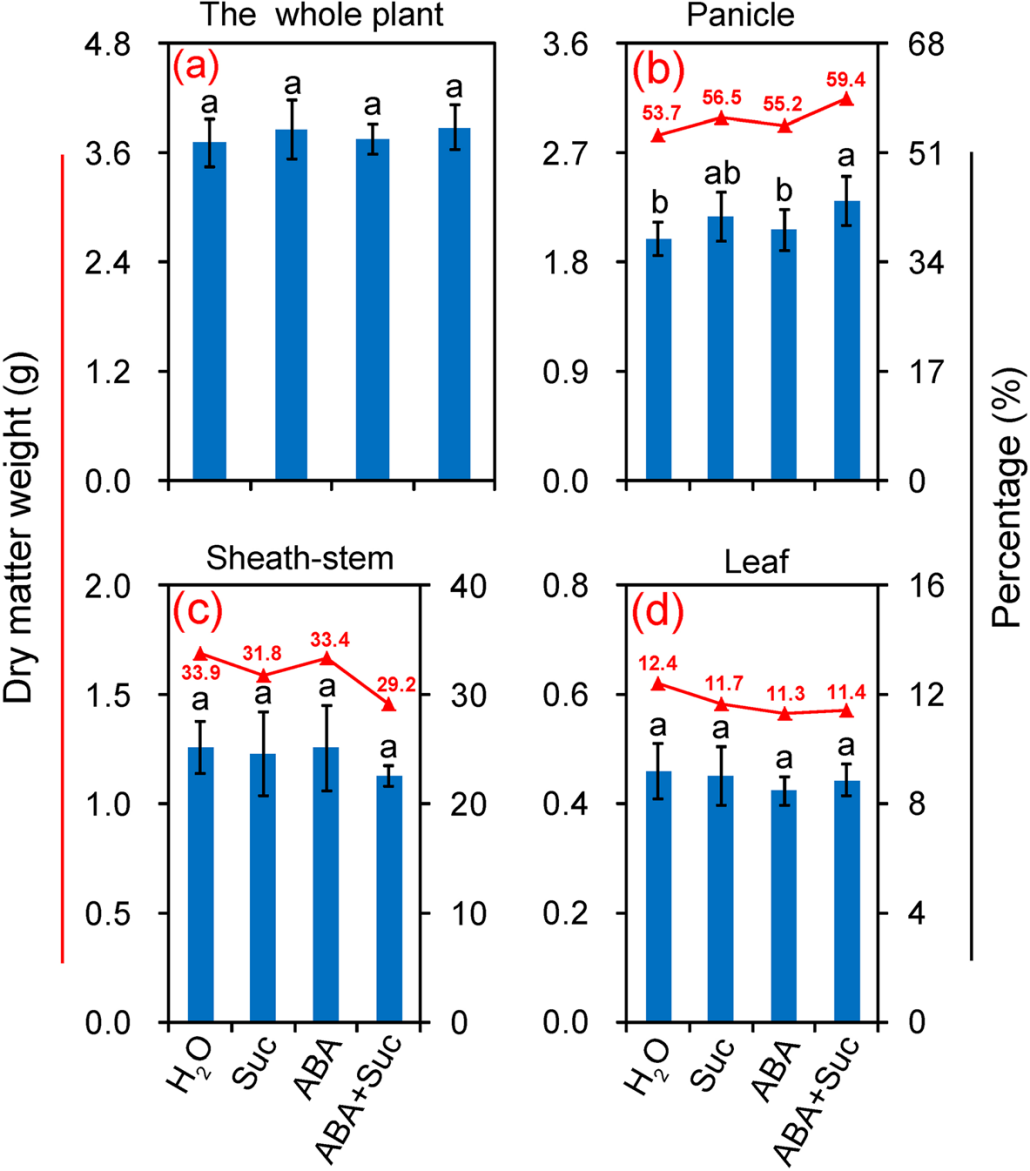
Dry matter accumulation and allocation in leaves, sheath-stems, and grains of rice under the sucrose, abscisic acid (ABA), and ABA plus sucrose treatments. The percentage represents the ratio of dry matter weight of panicles, sheath-stems or leaves to the whole plant. Vertical bars denote standard deviations (*n* = 3). Different letters indicate significant differences between chemical treatments (*P* < 0.05)

To investigate the effect of ABA and sucrose (either alone or in combination) on the assimilate distribution, the dry matter weights of leaves, sheath-stems, and grains were determined. As shown in Fig. 3a, no significant differences in total dry matter weights (whole plant) were found among the treatments (Fig. 3a). However, the panicle dry matter weight of plants treated with ABA plus sucrose was significantly higher than that of the control, where the ratio of the panicle dry matter weight to the whole plant was 59.4%, and percentages of 53.7, 56.5, and 55.2% were found in plants treated with H_2_O, sucrose, and ABA, respectively (Fig. 3b). Although no significant differences in dry matter weight of leaves and sheath-stems were found among the treatments (Fig. 3c and d), the highest value was found in the control, and the lowest values were found in the sheath-stems and leaves under the ABA plus sucrose and ABA treatments, respectively.

### Effect of ABA and sucrose on soluble sugar, starch, and non-structural carbohydrate contents of rice plants

**Fig. 4:**
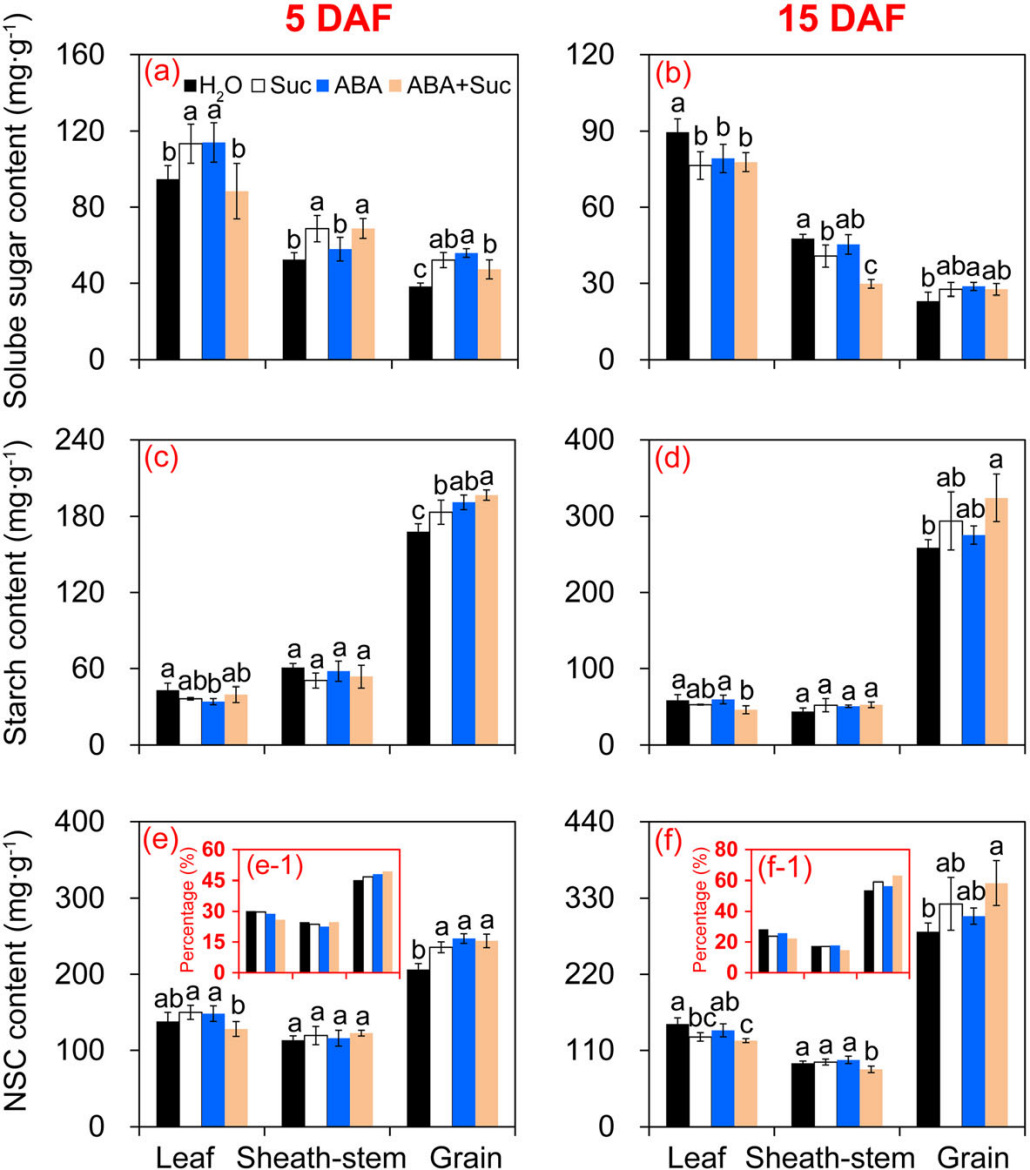
Changes of soluble sugar, starch, and non-structural carbohydrate (NSC) contents in leaves, sheath-stems, and grains of rice under the sucrose, abscisic acid (ABA), and ABA plus sucrose treatments. **a** and **b** Soluble sugar contents at 5 and 15 days after flowering (DAF), respectively; **c** and **d**, starch contents at 5 and 15 DAF respectively; **e** and **f**, NSC contents at 5 and 15 DAF, respectively. Vertical bars denote standard deviations (*n* = 3). Different letters indicate significant differences between chemical treatments (*P* < 0.05)

As shown in Fig. 4, changing patterns of soluble sugar, starch, and NSC were found in the leaves, sheath-stems, and grains among the treatments. Interestingly, the soluble sugar content of leaves in all treatments was higher than that of sheath-stems and grains (Fig. 4a and b). In contrast, the highest starch content was found in grains, which was significantly higher than that of leaves and sheath-stems (Fig. 4c and d). A similar pattern was found for NSC content, where the highest value was found in grains, followed by leaves and sheath-stems (Fig. 4e and f).

Soluble sugar contents in leaves of rice plants treated with ABA and sucrose were significantly higher than those of H_2_O and ABA plus sucrose at 5 days after flowering (DAF) (Fig. 4a), while in sheath-stems, the soluble sugar contents under the sucrose and ABA plus sucrose treatments were significantly higher than the contents under the H_2_O and ABA treatments. The highest value in grains was found in the ABA treatments, followed by sucrose and ABA plus sucrose treatments, which were all significantly higher than the control. At 15 DAF, the soluble sugar contents of leaves and sheath-stems under the control treatment tended to be higher than those of plants treated with ABA and sucrose alone or in combination. Furthermore, a pronounced increase in grain was found only in the ABA treatment compared to the control (Fig. 4b).

Starch in leaves and sheath-stems tended to be different among the treatments at 5 and 15 DAF (Fig. 4c and d). The highest starch content in grains at 5 DAF was found in the ABA plus sucrose treatment, followed by the ABA and sucrose treatments, which were all significantly higher than the control. However, no significant difference was found among these treatments except for plants treated with ABA plus sucrose, which was significantly higher than the control at 15 DAF.

No significant differences in NSC content of leaves were detected between the control and the ABA, sucrose, or ABA plus sucrose treatments at 5 DAF (Fig. 4e). However, the NSC content under the ABA plus sucrose treatment was lower than that under the ABA or sucrose treatments. No difference in NSC was observed in sheath-stems or grains among these four treatments except for NSC content in the control, which was significantly lower than the other treatments. At 15 DAF, the lowest NSC content of leaves and sheath-stems was found in plants treated with ABA plus sucrose, which was the lowest of all treatments (Fig. 4f). However, a significant increase was found in grains of plants treated with ABA plus sucrose compared to the control.

The highest NSC ratio of grains to the whole plant was found in the ABA plus sucrose treatment, followed by the ABA and sucrose treatments, while the lowest ratio was found in the control at 5 DAF (Fig. 4e–1). Similarly, the highest ratio of NSC in grains to the whole plant was detected in the ABA plus sucrose treatment, followed by the sucrose treatment, and the ABA treatment at 15DAF. Without exception, the lowest levels were found in the control (Fig. 4f–1).

### Effect of ABA and sucrose on sucrose, fructose, and glucose contents in rice

**Fig. 5:**
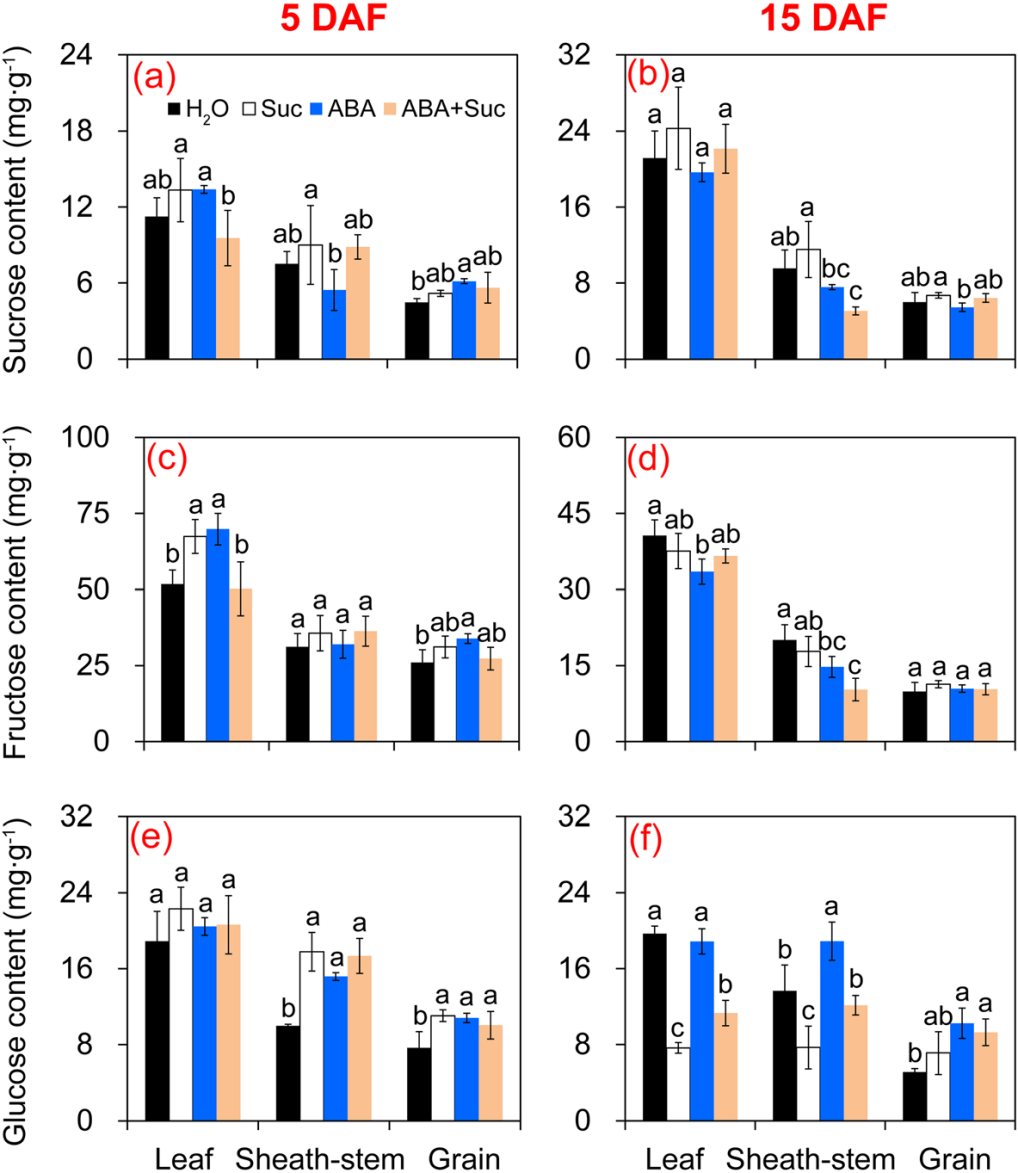
Changes of sucrose, fructose, and glucose in leaves, sheath-stems, and grains of rice under the sucrose, abscisic acid (ABA), and ABA plus sucrose treatments. **a** and **b** Sucrose contents at 5 and 15 days after flowering (DAF), respectively; **c** and **d**, fructose contents at 5 and 15 DAF, respectively; **e** and **f**, glucose contents at 5 and 15 DAF, respectively. Vertical bars denote standard deviations (*n* = 3). Different letters indicate significant differences between chemical treatments (*P* < 0.05)

To investigate the effect of ABA and sucrose on sucrose metabolism in leaves, sheath-stems, and grains, sucrose, glucose, and fructose contents were determined (Fig. 5). In general, the highest sucrose, glucose, and fructose contents were observed in leaves, followed by sheath-stems and grains. Different changing patterns of sucrose and fructose contents were found among the treatments at 5 and 15 DAF. However, ABA plus sucrose appeared to exert little effect on sucrose or fructose content, and no significant difference was detected between either treatments except for sheath-stems at 15 DAF (Fig. 5a–d). No significant difference in glucose content was found among the treatments in leaves at 5 DAF, whereas significant increases were found in sheath-stems and grains treated with sucrose and ABA alone or in combination compared to the control (Fig. 5e). At 15 DAF, the lowest glucose contents in leaves and sheath-stems were detected in plants treated with sucrose, which was significantly lower than that of the other treatments. However, the lowest glucose content in grains was found in control plants, which was lower than in plants treated with ABA and ABA plus sucrose (Fig. 5f).

### Effect of ABA and sucrose on key enzyme activities involved in grain filling

**Fig. 6:**
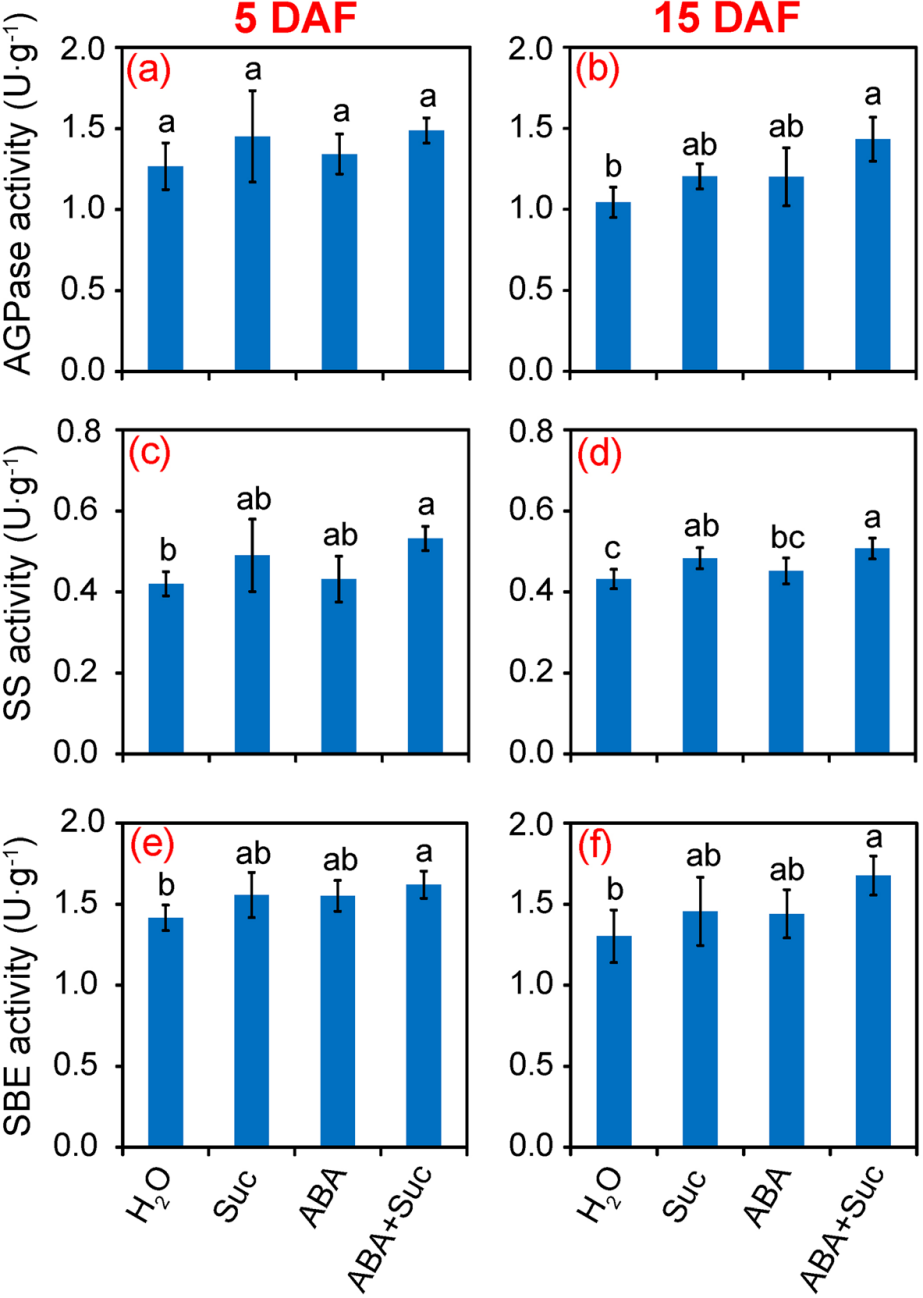
Effect of sucrose and abscisic acid (ABA) alone or in combination on the activities of adenosine diphosphate glucose pyrophosphorylase (AGPase), starch synthase (SS), and starch branching enzyme (SBE) in grains of rice. **a** and **b** AGPase activity at 5 and 15 days after flowering (DAF), respectively; **c** and **d**, SS activity at 5 and 15 DAF, respectively; **e** and **f**, SBE activity at 5 and 15 DAF, respectively. Vertical bars denote standard deviations (*n* = 3). Different letters indicate significant differences between chemical treatments (*P* < 0.05)

The activities of the key enzymes involved in grain filling of rice are shown in Fig. 6. No significant difference in AGPase activity was found in grains among the treatments at 5 DAF, while a significant increase was found under the treatment of ABA plus sucrose compared to the control at 15 DAF (Fig. 6a and b). A significant increase in starch synthase (SS) activity was only found in plants treated with ABA plus sucrose compared to the control at 5 DAF, while a significant increase was found under the sucrose and ABA plus sucrose treatments at 15 DAF (Fig. 6c and d). Similar patterns were found for starch branching enzyme (SBE) activity at 5 and 15 DAF, with a significant increase only in the ABA plus sucrose treatment compared to the control (Fig. 6e and f).

### Effect of ABA and sucrose on sugar transport and metabolism in leaves, sheath-stems, and grains of rice

**Fig. 7:**
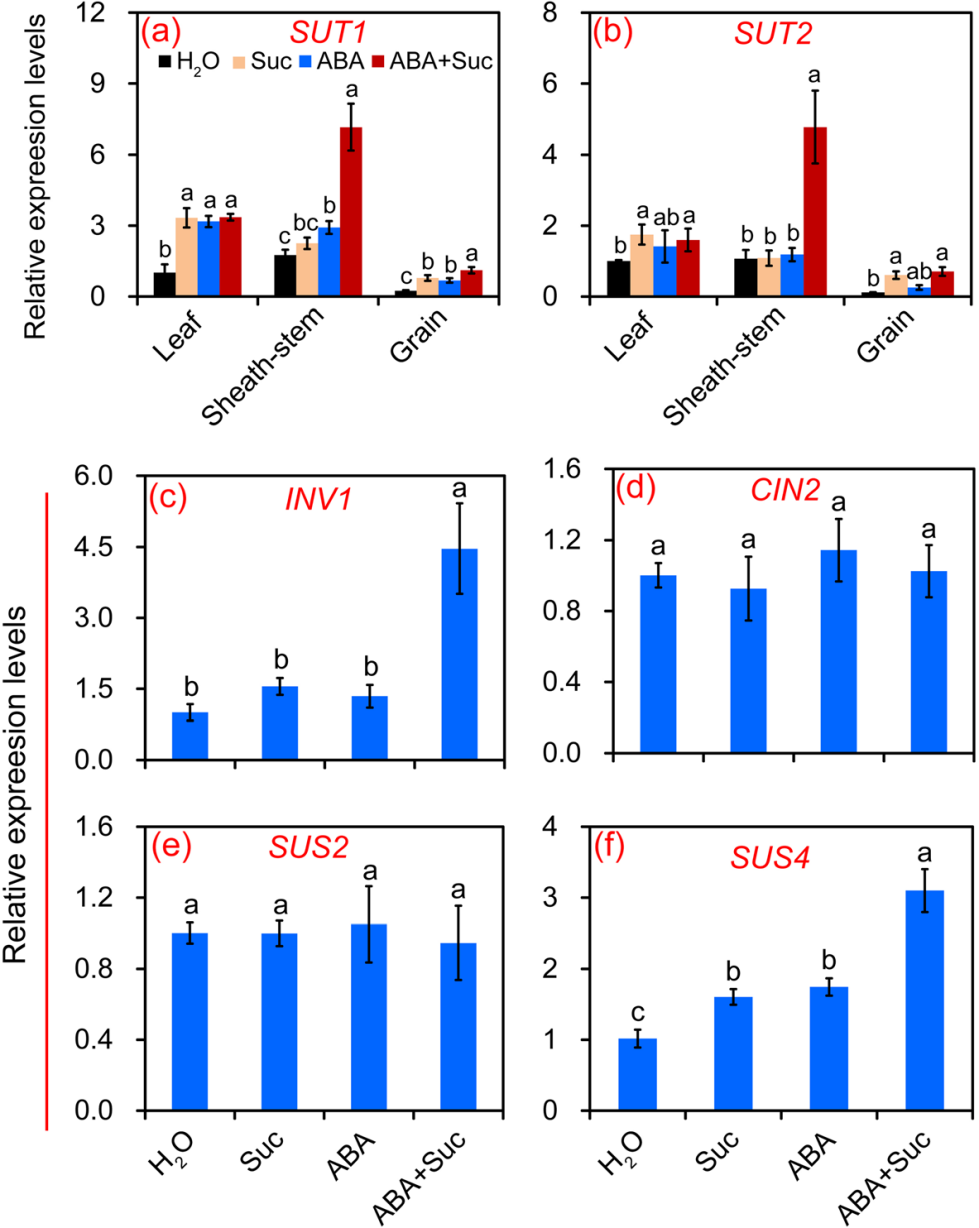
Effect of sucrose and abscisic acid (ABA) alone or in combination on the sucrose transport and metabolism of rice plants. **a** and **b** Effect of sucrose and ABA alone or in combination on the expression levels of *sucrose transporters* (*SUTs*), *SUT1* and *SUT2*, respectively, in leaves, sheath-stems, and grains of rice at 15 days after flowering (DAF); **c**–**f**, effect of sucrose and ABA alone or combination on the expression levels of *invertase1* (*INV1*), *cell-wall invertase2* (*CIN2*), *sucrose synthase* (*SUS*), *SUS2* and *SUS4* in grains of rice at 15 DAF. Vertical bars denote standard deviations (*n* = 3). Different letters indicate significant differences between chemical treatments (*P* < 0.05)

The relative expression levels of sucrose transporter (*SUT*) genes were determined at 15 DAF to investigate the effect of ABA and sucrose on sugar transport in leaves, sheath-stems, and grains. The relative expression levels of *SUT1* and *SUT2* in leaves increased significantly in response to sucrose or ABA alone or in combination, except for *SUT2* under the ABA treatment (Fig. 7a and b). Notable increases in relative expression of *SUT1* were found in sheath-stems of plants treated with ABA and ABA plus sucrose compared to the control (Fig. 7a), while a significant increase in *SUT2* was only found in the ABA plus sucrose treatment (Fig. 7b). In grains, the relative expression levels of *SUT2* increased significantly in response to sucrose or ABA alone or in combination, while such increases of *SUT1* were only found in plants treated with sucrose or ABA plus sucrose.

SUS and invertase (INV) are two main and important enzymes in higher plants that are responsible for metabolizing sucrose into glucose and fructose. Thus the relative expression levels of *INV1*, *cell-wall invertase2* (*CIN2*), *SUS2*, and *SUS4* were determined to investigate the effect of ABA and sucrose on sucrose metabolism in grains (Fig. 7c–f). No significant differences in the relative expression levels of *CIN2* or *SUS2* were found among the treatments. The relative expression level of *INV1* increased significantly only in plants treated with ABA plus sucrose compared to the control (Fig. 7c). However, the relative expression level of *SUS4* increased significantly in response to the sucrose and ABA alone or in combination, in particular the latter, compared to the control (Fig. 7f).

### Effect of ABA and sucrose on the trehalose metabolism in grains

**Fig. 8:**
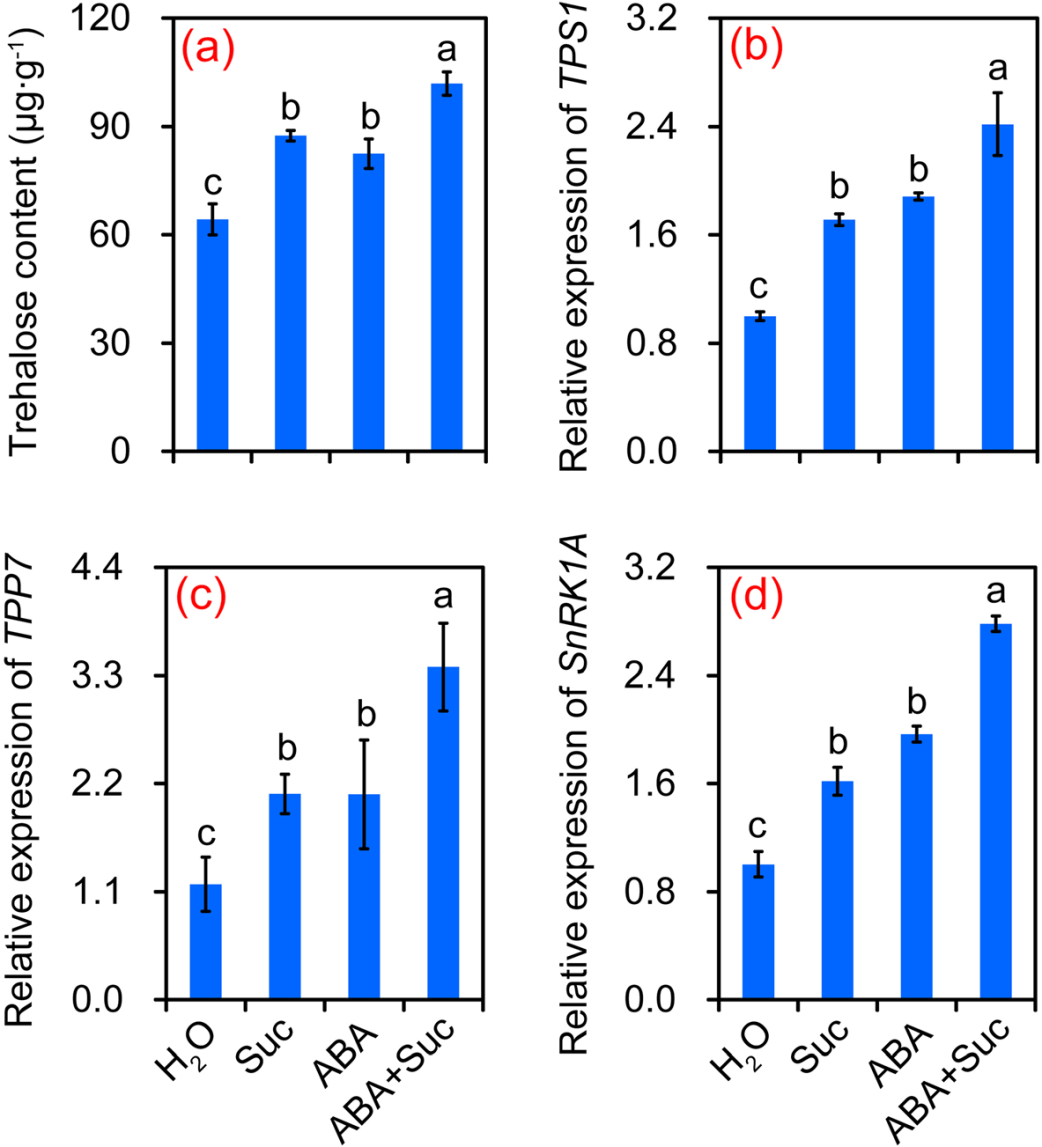
Effect of sucrose and abscisic acid (ABA) alone or in combination on trehalose metabolism in grains of rice. **a** Trehalose content under the sucrose, ABA, and ABA plus sucrose treatments; **b**–**d**, expression levels of *trehalose-6-phosphate synthase1* (*TPS1*), *trehalose-6-phosphate phosphatase7* (*TPP7*), and *sucrose non-fermenting related protein kinase 1A* (*SnRK1A*), respectively, under the sucrose, ABA, and ABA plus sucrose treatments. Vertical bars denote standard deviations (*n* = 3). Different letters indicate significant differences between chemical treatments (*P* < 0.05)

Trehalose metabolism, which can be affected by both ABA and sucrose, was also involved in the grain-filling of rice; thus, trehalose metabolism in grains was determined. As shown in Fig. 8a, the highest trehalose content was found in plants treated with ABA plus sucrose, followed by the sucrose and ABA treatments, which were all significantly higher than the control. A similar pattern was found in the expression levels of *trehalose-6-phosphate synthase1* (*TPS1*), *trehalose-6-phosphate phosphatase7* (*TPP7*), and *SnRK1A*, in which the highest levels were found in the ABA plus sucrose treatment, followed by the sucrose and ABA treatments, which were all significantly higher than the control (Fig. 8b–d).

### Effect of ABA and sucrose on phytohormone contents in leaves, sheath-stems, and grains of rice

**Fig. 9:**
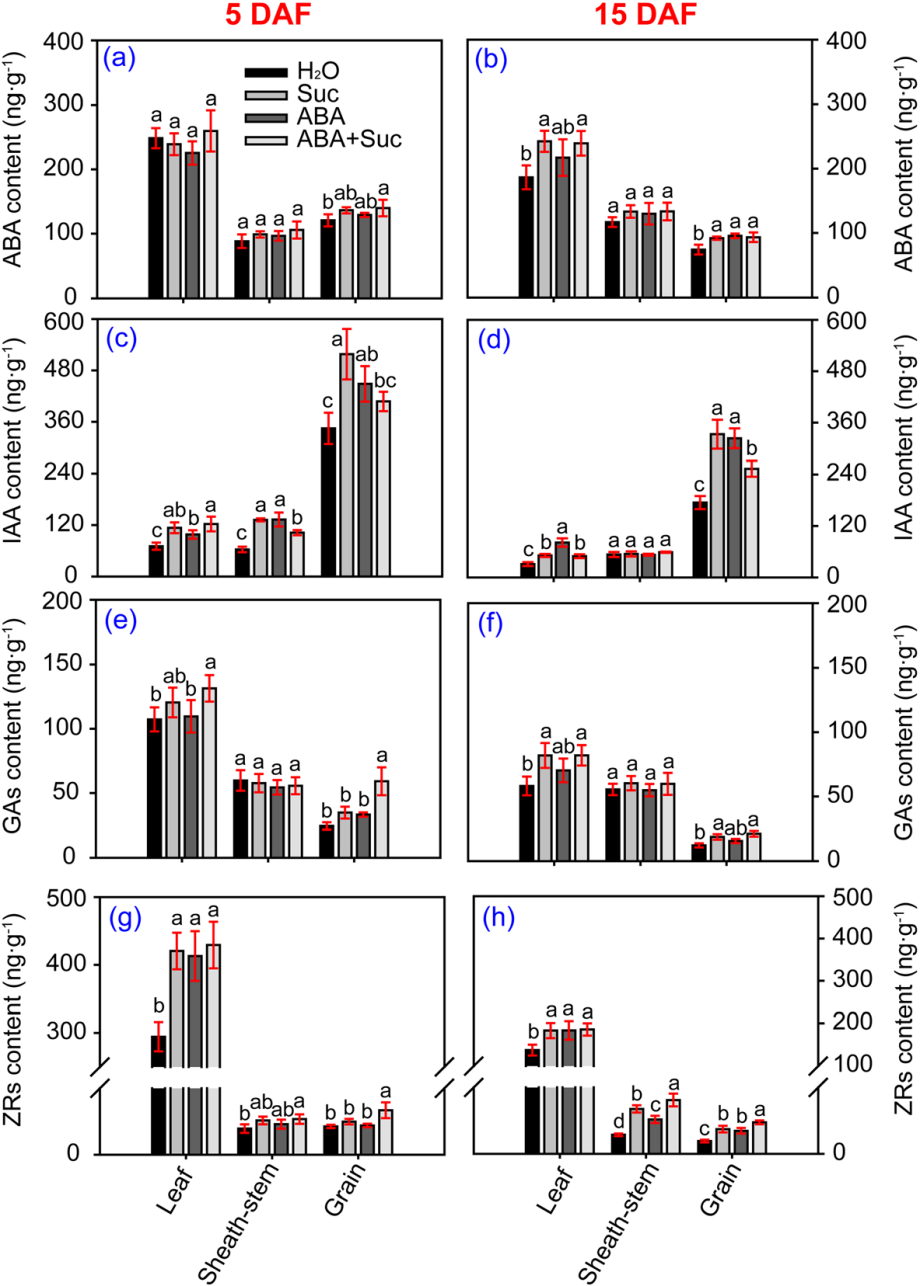
Effect of sucrose and abscisic acid (ABA) alone or in combination on endogenous phytohormone contents in leaves, sheath-stems, and grains of rice. **a** and **b** ABA contents at 5 and 15 days after flowering (DAF), respectively; **c** and **d**, indole acetic acid (IAA) contents at 5 and 15 DAF, respectively; **e** and **f**, gibberellic acids (GAs) contents at 5 and 15 DAF, respectively; **g** and **h**, zeatin ribosides (ZRs) contents at 5 and 15 DAF, respectively. Vertical bars denote standard deviations (*n* = 3). Different letters indicate significant differences between chemical treatments (*P* < 0.05)

Different patterns of phytohormones were detected in leaves, sheath-stems, and grains of rice plants when sprayed with ABA, sucrose, and ABA plus sucrose (Fig. 9). However, the highest contents of ABA, GAs, and zeatin ribosides (ZRs), were found in leaves, followed by sheath-stems, while the lowest content was found in grains. In contrast, the highest indole acetic acid (IAA) content was found in grains, followed by leaves, and sheath-stems.

No significant difference in ABA was detected among the treatments at 5 DAF except for the control treatment in grains, which was significantly lower than that of the ABA plus sucrose treatment (Fig. 9a). However, the ABA content in leaves and grains increased significantly in response to ABA, sucrose, and ABA plus sucrose compared to the control except in leaves in the ABA treatment (Fig. 9b). IAA increased significantly in response to ABA, sucrose, and ABA plus sucrose except in sheath-stems at 15 DAF, whereas no significant difference was found among the treatments (Fig. 9c and d). Interestingly, the highest IAA content in grains was detected in plants treated with sucrose at 5 and 15 DAF, which was significantly higher than that of plants sprayed with ABA plus sucrose. Significant increases in GAs contents were detected in leaves and grains under the ABA plus sucrose treatment at 5 DAF compared to the control, and a significant increase was found in plants treated with sucrose and ABA plus sucrose at 15 DAF (Fig. 9e and f). The ZRs contents in leaves increased significantly in response to sucrose, ABA, and ABA plus sucrose compared to the control at both 5 and 15 DAF (Fig. 9g and h). Significant increases in ZRs contents were only found in sheath-stems and grains in the ABA plus sucrose treatment at 5 DAF, whereas such effects were observed in the sucrose, ABA, and ABA plus sucrose treatments at 15 DAF. Accordingly, the greatest increase was found in plants treated with ABA plus sucrose compared to the control.

## Discussion

ABA and sucrose are important molecular signaling components that are involved in regulating plant growth and development [30, 31]. The cross-talk signaling between ABA and sucrose has been reported to regulate fruit ripening in tomato, strawberry, and maize [26, 32]. Such effects were also found in rice, where grain yield and quality of rice was significantly improved by ABA plus sucrose (Fig. 1).

The increase of grain yield in response to ABA plus sucrose was mainly ascribed to the higher seed-setting rate, where about a 15.7% increase was found (Fig. 1a). Notably, pollen tube elongation of the pistil was the determining factor for the seed-setting rate, as no significant differences in pollen tube germination on the stigma were found among the treatments (Fig. 2b). However, a greater number of longer pollen tubes were observed to have elongated to the ovary of plants treated with ABA plus sucrose at anthesis (Fig. 2 c and d). This observation suggests that sucrose plus ABA promotes pollen tube elongation into the ovary; thus, enhancing spikelet fertility. Indeed, heat stress inhibits elongation of the pollen tube into the ovary by impairing auxin homeostasis in pollinated pistils, which results in sterile spikelets [33]. However, in this study, auxin might not be the determining factor in this process, although this hormone plays a key role in pollen tube elongation in plants [34–37]. The highest auxin level was found in plants treated with sucrose at 5 DAF, while no significant difference was found between the control and sucrose plus ABA treatment (Fig. 9c). In contrast, marked increases in ABA, ZRs, and GAs levels were found in grains treated with sucrose plus ABA compared to the control (Fig. 9). GAs also functions in pollen tube elongation in plants [38–41]. However, the role of ABA and ZRs in pollen tube elongation of the pistil has rarely been investigated. Therefore, GAs might be the main regulatory factor in the process of pollen tube growth and embryo development caused by ABA plus sucrose, yet the underlying mechanism remains unclear.

The source-sink relationship is important for grain yield and quality of rice [42]. This relationship can be affected by environmental stress, where the expression levels of *SUT* in sheath-stems and grains are significantly inhibited at anthesis [4]. In this study, significant increases in the ratio of dry matter weight and NSC of the panicle to the whole plant were found in plants treated with sucrose plus ABA (Figs. 3 and 4). This finding suggests that improving the source-sink relationship might mainly contribute to the increase in grain yield and quality by sucrose plus ABA under such conditions. Sucrose or ABA alone increases assimilation in the grains from sheath-stems and leaves under extreme heat stress [4, 10]. However, higher ABA or sucrose inhibited the transport of assimilates; thus, reducing grain yield (Additional file 1: Figure S1). Therefore, crosstalk-signaling should be enhanced by ABA and sucrose, rather than only enhancing the concentration of each other [43–45]. The results of the present study indicate that sucrose enhanced the ABA level in plants, but no significant differences in sucrose content or ABA were found among the sucrose, ABA, or ABA plus sucrose treatments (Figs. 5 and 9). It has been reported that exogenous sucrose acts as a signal transducer and promotes ripening of strawberry fruit by altering the gene expression of enzymes involved in ABA synthesis and affecting endogenous ABA content [26]. Additionally, crosstalk between sucrose and ABA could stimulate the activity of cellular signal transduction pathways by inducing the expression of several important genes involved in this process [9, 46, 47].

**Fig. 10:**
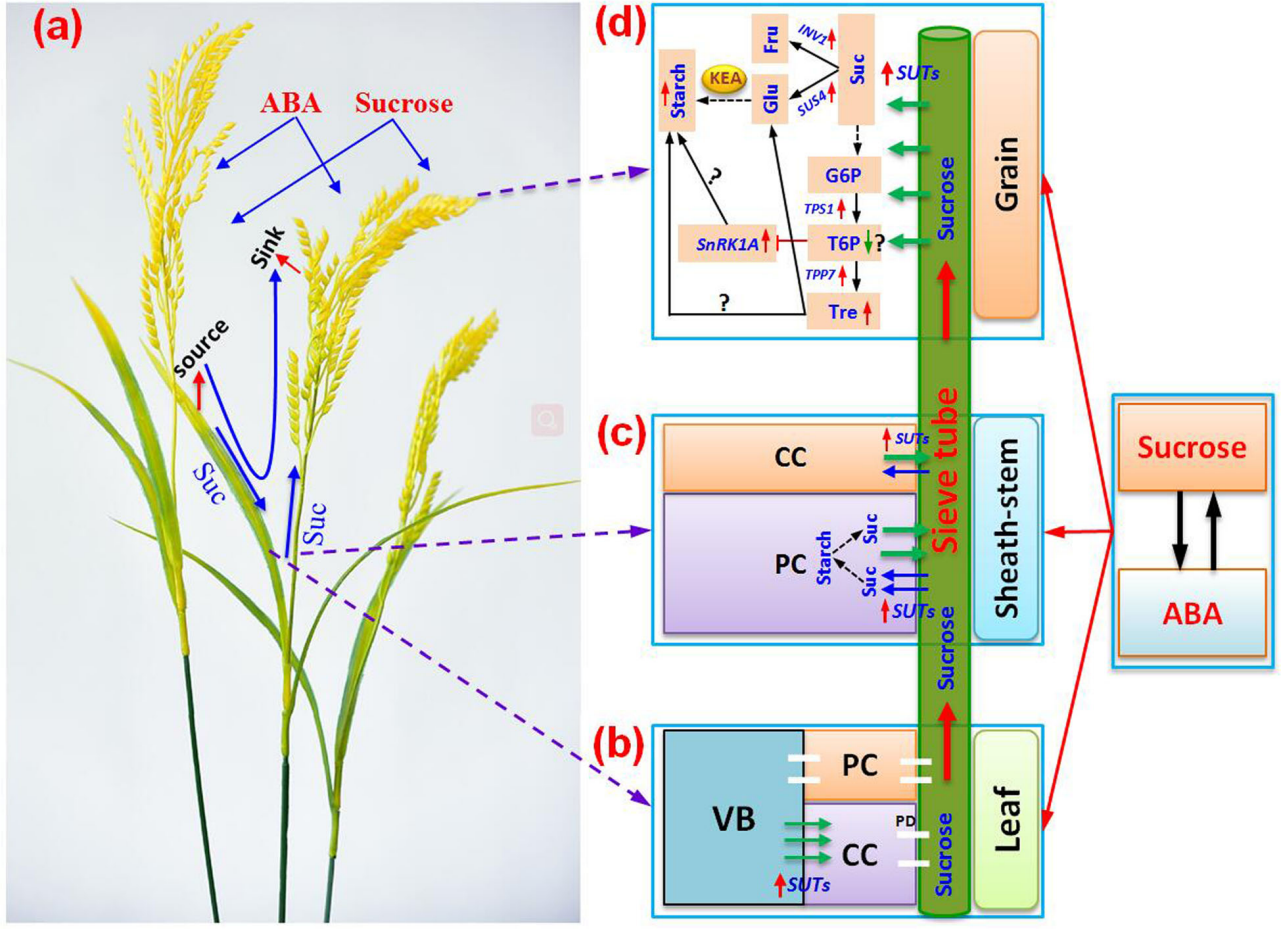
Descriptive model of the crosstalk between sucrose and abscisic acid (ABA) functions in the source-sink relationship of rice plants. ABA synergized with sucrose, which significantly improved the source-sink relationship of rice plants. In this process, the *sucrose transporters* (*SUTs*) genes, *SUT1* and *SUT2*, were induced by ABA plus sucrose in leaves, which were mainly responsible for sucrose loading from the vascular bundles (VB) to the companion cells (CC) of leaves, and then transported to the sheaths and panicles through the plasmodesma (PD); sucrose in sheath-stems was unloaded by *SUTs* and stored in the CC or parenchymatous cells (PC) in the form of starch, while it was more loaded and transported to the grains through the sieve tubes by ABA plus sucrose in this study. In grains, the synergy between ABA and sucrose significantly increased starch content by enhancing the expression of *invertase1* (*INV1*) and *sucrose synthase4* (*SUS4*) as well as the key enzyme activity (KEA) of grain filling. Additionally, trehalose-6-phosphate (T6P) content was deduced to decrease in response to ABA plus sucrose as a greater increase in gene expression of *trehalose-6-phosphate phosphatase7* (*TPP7*) than *trehalose-6-phosphate synthase1* (*TPS1*), and an increase in *sucrose non-fermenting related protein kinase 1A* (*SnRK1A*) expression. Thus, trehalose content increased and trehalose metabolism was improved by ABA plus sucrose to regulate starch synthesis and assimilate allocation, where T6P and SnRK1A were the main mediators. Fru, fructose; Glu, glucose; G6P, glucose-6-phosphate; Suc, sucrose; Tre, trehalose; the arrows ‘→’ and “” indicate direct and indirect induction in D, while ‘┤’ indicates inhibition

As the photosynthetic product of the chloroplast tissue in plants, sucrose is largely responsible for carbohydrate transport from source organs to metabolic organs through long-distance movement in the sieve elements of the phloem [48–50]. This process includes sucrose loading, transport, and unloading via apoplast or symplast pathways under catalysis by multiple proteins, such as SUTs and their family, SS, and INV for sucrose to traverse cell membranes [51]. According to the present data, although ABA plus sucrose enhanced sucrose loading in leaves and unloading in grains, it might mainly function in sucrose transport in sheath-stems and in sucrose metabolism in grains (Fig. 10). This is because of the significant increase by several orders of magnitude in the expression levels of *SUT1* and *SUT2* in sheath-stems under the sucrose plus ABA treatment (Fig. 7). As reported, sucrose transport from source to sink can be affected by external environmental conditions, and temperature stress induces impaired source-to-sink transport; thus, hindering sucrose delivery [52]. Improvement of the phloem sucrose transport ratio has also been identified as a potential target for enhancing plant productivity [53]. It has been reported that ABA induces the expression of sugar transporter and amylase genes; thus, increasing soluble sugar accumulation and improving fruit quality [54]. Interestingly, no differences in hormone content were found among the treatments in sheath-stems except for ZRs, where a significant increase was found in plants treated with sucrose plus ABA (Fig. 9h). Therefore, ZRs might be mediators of the significant increase in *SUT1* and *SUT2* expression levels in sheath-stems by ABA plus sucrose, as cytokinins have been reported to enhance sucrose transport in plants [55, 56]. However, how sucrose plus ABA affects ZRs contents in plants remains unclear.

ABA plus sucrose also enhanced sucrose metabolism in rice grains, where significant increases in the expression levels of *INV1* and *SUS4* were detected (Fig. 7c and f). This finding was partially consistent with previous results indicating that ABA induces the expression of *INV1* and *SUS4* in spikelets under heat stress at the pollen mother cell meiosis stage of rice [10]. Additionally, exogenous sucrose significantly increases sucrose transporter activity [57]. Interestingly, a similar change in ZRs was also found in grains, where the highest ZRs contents was found in the sucrose plus ABA treatment, followed by the sucrose or ABA treatments, which were significantly higher than the control (Fig. 9). This finding suggests that ZRs were also involved in sucrose metabolism in grains in response to sucrose plus ABA (Fig. 9h).

The trehalose pathway is a central system that integrates growth and development via the supply of sucrose. The accumulation of trehalose in plants confers high tolerance to different abiotic stresses [58, 59]. In this study, a significant increase in trehalose content was detected in plants treated with sucrose or ABA alone or their combination, and particularly for the ABA plus sucrose treatment (Fig. 8). This finding suggests that the synergy between ABA and sucrose enhanced trehalose metabolism and improved the source-sink relationship. Additionally, ABA plus sucrose significantly increased the expression levels of *TPS1* and *TPP7* in grains (Fig. 8), which was mainly responsible for the synthesis of trehalose and enhances tolerance to stress [58, 60]. However, the role of trehalose in signaling remains controversial, as trehalose-6-phosphate (T6P), rather than trehalose, has been suggested to mainly function in pathways that regulate sugar allocation between the source and sink in plants [59]. T6P is a central sugar signal in plants that regulates sucrose use and allocation, underpinning crop growth and development [61, 62]. The significant enhancement in T6P levels via chemical intervention increases both yield and resilience [63]. However, the levels of T6P could not be determined in this study; therefore, its role in assimilate allocation in response to sucrose and ABA alone or their combination could not be directly evaluated. However, a significant increase in *SnRK1A* expression level was detected in grains treated with sucrose and ABA alone or combination compared to the control (Fig. 8d). This finding suggests that T6P level decreased in response to ABA plus sucrose, as *SnRK1A* expression can be inhibited by T6P [59, 62, 64]. Additionally, a greater increase in expression level in *TPS1* than *TPP7* was observed in grains treated with sucrose and ABA alone or in combination (Fig. 8b and c). The former is responsible for synthesis of T6P, while the latter reduces the T6P level for the synthesis of trehalose [58, 61]. The higher TPP/TPS ratio could result in a lower T6P level. Interestingly, it has been reported that over-expression of *TPP* increased both kernel set and the harvest index by reducing the T6P concentration, and increasing the concentration of sucrose in ear spikelets [61]. Thus, it was considered that enhanced TPP7 activity may increase the sink strength in proliferating heterotrophic tissues by indicating low sugar availability and increasing T6P turnover. This enhances starch mobilization to drive the growth kinetics of the germinating embryo and elongating coleoptile, which consequently enhances anaerobic germination tolerance [58]. Thus, the sucrose plus ABA treatment reduced the T6P level in grains to improve the source-sink relationship of rice by inducing the expression of *SnRK1* (Fig. 10d). This process was activated in response to declining energy supply (e.g., during stress); thus, triggering activation of catabolism and the repression of energy-consuming anabolic processes and growth [18]. However, *SnRK1* can either be directly or indirectly regulated by sucrose or ABA alone in plants independent of the presence of stress. Therefore, more studies should focus on the relationship among sucrose, ABA, T6P, trehalose, and SnRK1 in plants, as well as their effects on sugar allocation.

## Conclusion

In summary, the synergy between sucrose and ABA significantly increased grain yield and quality of rice by improving the source-sink relationship (Fig. 10). The sucrose transporter genes (*SUT1* and *SUT2*) were the main factors in this process, particularly in sheath-stems and grains (Fig. 10), where significant increases in *SUT1* and *SUT2* expression levels were found. The ZRs contents, key enzymatic activities such as AGPase, SS, and SBE, as well as the expression levels of *INV1* and *SUS4* increased significantly in grains in response to sucrose plus ABA. This finding suggests that ABA plus sucrose enhanced sucrose metabolism and thus regulated starch synthesis. Additionally, significant increases in trehalose contents, and the expression levels of *TPS1*, *TPP7*, and *SnKR1A* were also detected in grains in response to the ABA plus sucrose treatment, indicating that this crosstalk signaling could affect starch synthesis by regulating *SnRK1A* to improve the source-sink relationship (Fig. 10). In summary, a synergistic effect was found between sucrose and ABA in rice plants, which significantly increased grain yield and quality of rice by improving the allocation of assimilates mainly through sucrose and trehalose metabolism.

## Methods

### Plant materials and growth conditions

The Zhefu802 *indica* variety, which is conventionally cultivated for rice production in China, was chosen for the current experiment from May to August. The plant materials were grown at the Fuyang experimental base of the China National Rice Research Institute in Hangzhou city, Zhejiang Province, China. The seeds were originally provided by the Institute of Nuclear Agronomy, Zhejiang Agricultural University and were soaked to hasten germination and then sterilized. They were washed, sprouted at 35°Cfor approximately 24 h in the dark until the length of the bud reached (but did not exceed) 0.5 cm, and then the seedlings were sown in paddy seedbeds. The 1-month old rice seedlings were transplanted in a plastic tank filled with soil from the field and grown in an artificial climate chamber with an automatic temperature control system to regulate the day/night temperature at 32/24 °C, respectively until maturity by adopting the micro plot trail under natural sunlight conditions. The aboveground parts of the rice plants (mainly the leaves and panicles) received chemical sprays of ABA (10 μM), sucrose (15 mM), or their combination (10 μM ABA + 15 mM sucrose) respectively at flowering and 10 DAF. The applied concentrations of chemicals were optimized and set according to the results of a preliminary experiment (Additional file 1: Figure S1). Chemicals containing 0.1% (v/v) Tween-20 as the surfactant were applied at 9:00 a.m. at a volume of 100 ml·m^− 2^. Deionized water was used as a control for spraying. Fresh samples including leaves, sheath-stems, and spikelets of rice plants were separated and sampled to determine carbohydrate contents, starch synthesis-related enzyme activities, and hormone contents at 5 and 15 DAF. They were instantly frozen in liquid nitrogen, and stored at − 80°Cuntil further analysis.

### Determination of dry matter weight, grain yield, and quality

Fresh leaves, sheath-stems, and panicles of rice were sampled, and their dry matter weights were determined by separating these parts from mature plants, and drying them at 75 °C for 3 d until they reached a constant weight. Grain yield was determined from harvested plants grown within a 1 m^2^ area of each plot. Yield components, including seed-setting rate and kernel weight were determined from three single hills of each replication. Seed-setting rate was calculated as the ratio of filled grains, selected by air separation to total grain numbers. Grain samples of more than 250 g were delivered to the quality inspection center of the China National Rice Research Institute to measure the milled rice ratio, the head milled rice ratio, and amylose content.

### Observations of pollen tube germination, elongation, and grain size

Fifty stigmas from flowering spikelets were collected 2 h after flowering 1 day after the chemical application. The samples were fixed in Carnoy’s fixing reagent containing chloroform-ethanol-acetic acid (volume ratio of 3:6:1) for at least 24 h. They were then washed with deionized water, cultivated in 10 M NaOH for 8 min at 56 °C, and dyed with 0.1% (m/v) aniline blue solution for 24 h [33]. A fluorescence microscope (DM4B, Leica, Wetzlar, Germany) was used to observe pollen germination on the stigma, and elongation of the tube to the ovary and stigma were screened at an optical wavelength of 350 nm. The pollen tube elongation ratio was defined as the percentage of the number of spikelets that successfully finished the process of pollen tube elongation to the ovary.

Grains harvested at the maturity stage were dehulled and photographed. The dimensions of each grain were measured for each replicate treatment using Image J software (Version 1.46, National Institutes of Health, Bethesda, MD, USA).

### Soluble sugars, starch, and NSC contents

Total soluble sugar contents were determined according to a previously described method [10]. About 0.1 g powder of dried and disrupted plant tissues were saturated in 10 ml of 80% (v/v) ethanol in an 80 °C water bath for 30 min. After cooling, the solution was centrifuged at 2000 rpm for 15 min, and this extraction process was repeated three times. The supernatants were combined, filtered, and added to 0.2% (m/v) anthrone, prepared with sulfuric acid and kept in a boiling water bath for 15 min. Absorbance was recorded at 485 nm with a visible spectrophotometer (Model 722S, Lengguang Technology, Shanghai, China). The starch-containing residue was dried, boiled with deionized water, and fully blended. Perchloric acid of 9.2 M and 4.6 M was added to the cooled mixture and continuously shaken. Then, the supernatant was collected after centrifugation, and starch content was determined following the method used to estimate total soluble sugar. NSC content was calculated as total soluble sugar plus starch.

### Sucrose, fructose, glucose, and trehalose contents

Sucrose, glucose, and fructose in leaves, grains, and sheath-stems of rice were extracted using the same method as described for extracting soluble sugar [10]. Each 0.9 ml of extracted supernatant was blended with 0.1 ml of 2 M NaOH to measure sucrose content, and kept in boiling water for 10 min to eliminate impurities. Then, 1.0 ml of 0.1% (m/v) resorcinol dissolved in 95% (v/v) ethanol and 3 ml of 10 M HCl were added. The mixture was kept in a water bath at 80 °C to actuate the chemical reaction. After 1 h, absorbance of the compound liquid was determined at 500 nm. Another 1 ml of extracted supernatant was successively added with 0.6 ml of 0.1% (m/v) resorcinol and 2.4 ml 10 M HCl to determine fructose content. Absorbance at 480 nm was recorded after the catalytic reaction of the solution in a water bath at 80°Cthat lasted 30 min. Each 4.0 ml of the reaction unit consisting of 30 μM O-dianisidine dihydrochloride, 10 mg of 20 U·L^− 1^ horseradish peroxidase, and 20 U·L^− 1^ of glucose oxidase (pH 5.5, dissolved in 0.1 M acetic acid buffer) was prepared to determine glucose content. The enzyme was warmed in a water bath, and 2 ml of extracted supernatant was added once the temperature reached equilibrium at 30 °C. The reaction was stopped by adding 8.0 ml of 5 M sulfuric acid after 5 min. Absorbance was recorded at 505 nm.

Trehalose content was determined as described by Wang and Ge [65] using high-performance ion exchange chromatography (ICS-600, Thermo Fisher Scientific Inc., Waltham, USA). About 1 g of fresh grains were homogenized in liquid nitrogen, extracted in 10 ml deionized water, and incubated in boiling water for 10 min. After centrifugation at 10,000 rpm for 10 min, the supernatant was transferred to a clean tube and passed through a 0.45 μm microfiltration membrane. Samples of 50 μl were injected into an analytical column and a protected column with sizes of 4 mm × 250 mm and 4 mm × 50 mm, respectively (CarboPac MA1, Thermo Fisher Scientific Inc., Waltham, USA). Samples were eluted from the column by an assorted pump at 30 °Cwith sodium hydroxide at a flow rate of 1.0 ml·min^− 1^. Trehalose was detected by a Dionex ICS-4000 ED detector and its content was further analyzed.

### Determining endogenous phytohormone contents

Aliquots (0.2 g) of fresh leaves, grains, or sheath-stems sampled at 5 and 15 DAF were ground in 80% (v/v) methanol containing 1 mM butylated hydroxytoluene as an antioxidant and extracted in an ice bath. The digestion was kept at 4 °C for at least 4 h; then, the supernatants were collected after centrifugation at 4000 rpm for 10 min. The samples were vacuum dried to remove the methanol. The samples were then added to 1 ml of 10 mM pH 7.4 phosphate buffered solution containing 0.1% (v/v) Tween-20 and 0.1% (m/v) gelatin and were fully dissolved. ABA, IAA, GAs, and ZRs contents were detected via enzyme-linked immunosorbent assay kits according to the manufacturer’s instructions (China Agricultural University, Beijing, China).

### Determination of key enzymes involved in starch synthesis

AGPase, SS, and SBE were extracted as described by Zhang et al. [66]. In brief, approximately 40 dehulled grains were fully ground in 5 ml of 0.1 M pH 8.0 Tricine-NaOH, containing 10 mM magnesium chloride, 2 mM EDTA, 50 mM β-mercaptoethanol, 12% (v/v) glycerol, and 5% (w/v) PVP40 with a mortar in an ice bath. Immediately after centrifugation at 15,000 rpm for 10 min at 4 °C, the supernatant of the extracted fragments was used to determine enzyme activities. AGPase, SBE, and SS activities were determined according to Nakamura et al. [67] and Schaffer and Petreikov [68] using ultraviolet spectrophotometry (UV-2600, Shimadzu, Kyoto, Japan). The activities of these enzymes were expressed as U·g^− 1^ fresh weight of grain.

### RNA extraction and qRT-PCR analysis

Total RNA was extracted from 0.1 g of leaves, grains, and sheath-stems of rice plants at 15 DAF using the Plant RNA Reagent (Invitrogen, Carlsbad, CA, USA). First-strand cDNA was synthesized from RNA using the Quantscript RT Kit (Qiagen, Hilden, Germany). Relative expression levels of target genes, including *SUT1* and *SUT2*, which are involved in sucrose transport [69, 70]; *INV1*, *CIN2*, *SUS2*, and *SUS4* involved in sucrose conversion [71–73]; and *TPS1*, *TPP7*, and *SnRK1A* involved in trehalose metabolism [74] were detected by the Step One Plus Real-time PCR System (Thermo Fisher Scientific, USA) using the Power Up SYBR Green Supermix (Applied Biosystems, Foster City, CA, USA). Primers were designed based on the anticipated size of the amplification products (150–250 bp) as listed in Additional file 2: Table S2. Primer Premier 5.0 software (Premier, Ottawa, ON, Canada) was used to complete the design work [75]. A three-step method for the PCR reaction was adopted, and the procedure was carried out as described previously [76]. The relative transcript levels of the target genes were analyzed by the 2^−ΔΔCT^ method and expressed as means of triplicate experiments.

### Statistical analysis

Data were processed with SPSS 11.5 software (IBM Corp, Armonk, NY, USA) to determine the least significance difference at *P* ≤ 0.05 by one-way analysis of variance. The data and standard errors in the figures represent mean values of three repetitions.

## Supplementary information

**Additional file 1: Figure S1.** Effect of sucrose and abscisic acid (ABA) on grain yield of rice. Vertical bars represent standard deviations (*n =* 3). Different letters indicate significant differences between chemical treatments (*P* < 0.05). Chemicals, including sucrose, ABA, and fluridone (ABA inhibitor) were dissolved in deionized water and sprayed onto the rice plants. The sucrose concentrations were: 0, 3, 15, 75, and 375 mM; the ABA concentrations were: 0, 1, 10, and 100 μM, and the fluridone concentrations were: 10 and 100 μM.

**Additional file 2: Table S1.** Effect of sucrose and abscisic acid (ABA) alone or in combination on the rice quality; **Table S2.** Primer sequences used in the quantitative RT-PCR.

## Data Availability

The datasets used and/or analyzed during the current study are available from the corresponding author on reasonable request.

## Abbreviations

ABA: Abscisic acid
AGPase: Adenosine diphosphate glucose pyrophosphorylase
CIN: Cell-wall invertase
DAF: Days after flowering
GAs: gibberellic acids
IAA: Indole-3-acetic acid
INV: Invertase
NSC: Non-structural carbohydrate
SBE: Starch branching enzyme
SnRK1: Sucrose non-fermenting related protein kinase 1
SS: Starch synthase
SUS: Sucrose synthase
SUT: Sucrose transporter
T6P: Trehalose-6-phosphate
TPP: Trehalose-6-phosphate phosphatase
TPS: Trehalose-6-phosphate synthase
ZRs: Zeatin ribosides
