# Supplementary figures and images for: Abscisic acid synergizes with sucrose to enhance grain yield and quality of rice by improving the source-sink relationship
Source: BMC Plant Biol. 2019 Nov 27;19:525. doi: 10.1186/s12870-019-2126-y (PMC6882056; doi:10.1186/s12870-019-2126-y)

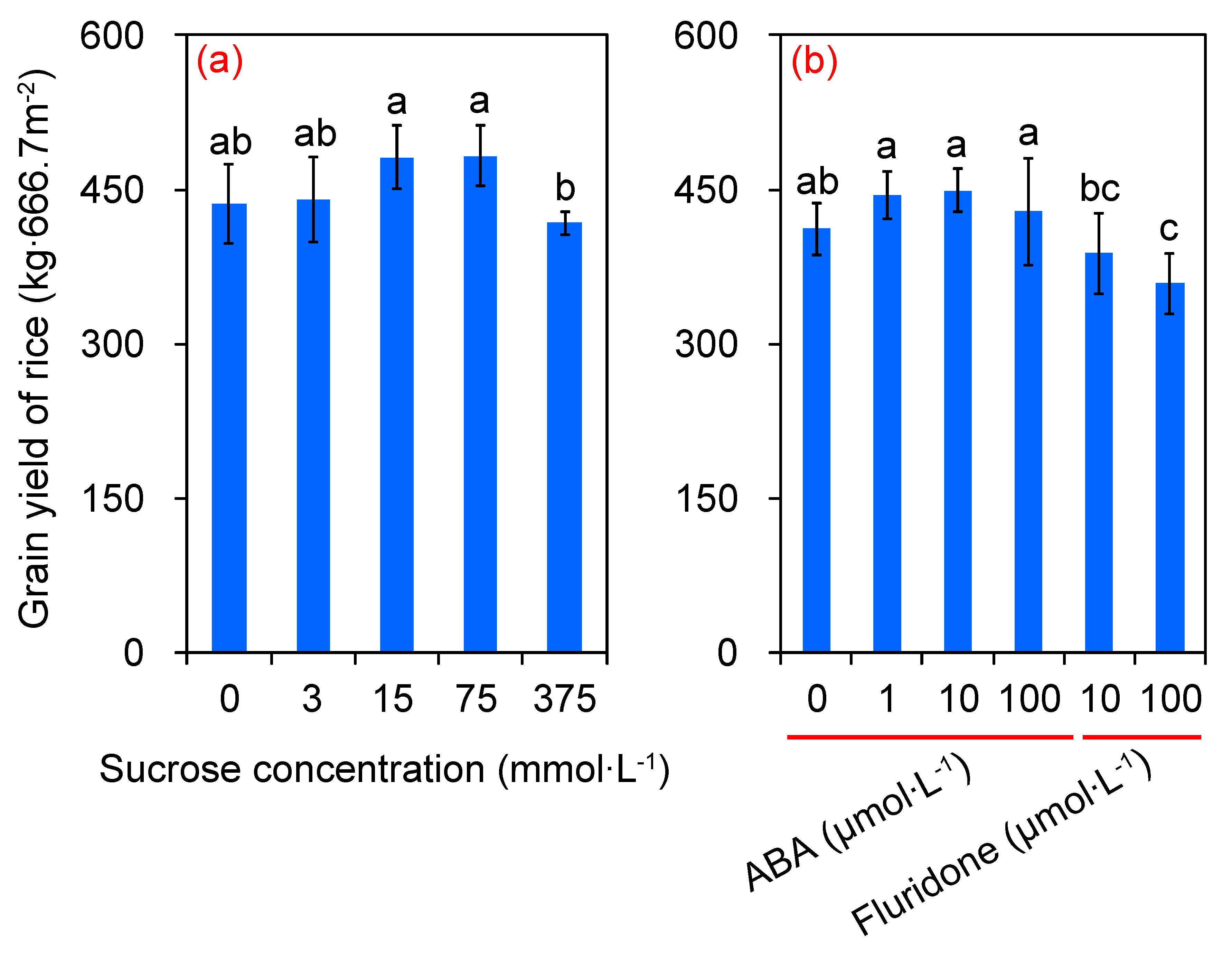

Supplement: Supplementary file 1 — Additional file 1: Figure S1. Effect of sucrose and abscisic acid (ABA) on grain yield of rice. Vertical bars represent standard deviations (n = 3). Different letters indicate significant differences between chemical treatments (P < 0.05). Chemicals, including sucrose, ABA, and fluridone (ABA inhibitor) were dissolved in deionized water and sprayed onto the rice plants. The sucrose concentrations were: 0, 3, 15, 75, and 375 mM; the ABA concentrations were: 0, 1, 10, and 100 μM, and the fluridone concentrations were: 10 and 100 μM. [file 12870_2019_2126_MOESM1_ESM.jpg]
