# Supplementary material for: Abscisic acid synergizes with sucrose to enhance grain yield and quality of rice by improving the source-sink relationship
Source: BMC Plant Biol. 2019 Nov 27;19:525. doi: 10.1186/s12870-019-2126-y (PMC6882056; doi:10.1186/s12870-019-2126-y)
Supplement: Supplementary file 2 — Additional file 2: Table S1. Effect of sucrose and abscisic acid (ABA) alone or in combination on the rice quality; Table S2. Primer sequences used in the quantitative RT-PCR. [file 12870_2019_2126_MOESM2_ESM.docx]

**Table S1** Effect of sucrose and ABA alone or in combination on the rice quality.

| Treatment | Brown rice  rate (%) | Chalky grain  rate (%) | Chalkiness  (%) | Transpare-  ncy | Ratio of length  to width (%) | Alkali  value | Gel consist-  ency (mm) |
| --- | --- | --- | --- | --- | --- | --- | --- |
| H_2_O | 78.4 a | 36.6 a | 13.1 a | 4 a | 2.23 a | 5 a | 64.1 a |
| Sucrose | 79.7 a | 38.5 a | 12.9 a | 4 a | 2.25 a | 5 a | 62.5 a |
| ABA | 76.5 a | 39.1 a | 11.4 a | 4 a | 2.20 a | 5 a | 66.3 a |
| ABA+Sucrose | 78.8 a | 37.3 a | 12.8 a | 4 a | 2.21 a | 5 a | 66.0 a |

The same letter within one column means no statistically significant difference among treatments (*P*＜0.05).

**Table S2** Primer sequences used in the quantitative RT-PCR

| Gene | cDNA number | Forwad(5'-3') | Reverse(5'-3') |
| --- | --- | --- | --- |
| *SUT1* | AK100027 | GGTGGCATGCTGCTATTGTA | GTTAGCTGTGCCAGGTCCAT |
| *SUT2* | AB091672 | TGGTCACTCAAGGACTTCCA | CACTGTACAGAACGGCGAGA |
| *INV1* | AF155121 | AAGAGCAGGGGTGTACAAG | AAGCTGTGAGTCTGTGGCTC |
| *CIN2* | EU095553 | AACATGTACCAGCCGACGTTTG | AAGCTCTCAACAACCGACCTGT |
| *SUS2* | AK072074 | GTGTGCTTGACACCATCCAC | CATGCGGAGACAGGATAACA |
| *SUS4* | AK102158 | GGCTGGGGTTTATGGCTTCT | TCTCATCACGTCGAGGACCT |
| *TPP7* | AK288009 | CGCCAAGGTCGAGAACAACAAG | CTTGCCCTTGTCCCACTTGATG |
| *TPS1* | AK072132 | GAAGAGCGGCCATCAGATTGTG | CAAGCTGCCACGATTTCCCATG |
| *SnRK1B* | AP003743.4 | CTGCGGCTGTGTTCTTTGAAATT | AGAAAGCCTCTTCAGATCCATCA |
